# Impact of Initial Operative Urgency on Short-Term Outcomes in Patients Treated with ECMO Due to Postcardiotomy Cardiogenic Shock

**DOI:** 10.3390/life12111872

**Published:** 2022-11-13

**Authors:** Borko Ivanov, Ihor Krasivskyi, Stephen Gerfer, Anton Sabashnikov, Mirko Doss, David Holzhey, Kaveh Eghbalzadeh, Christian Rustenbach, Elmar Kuhn, Parwis Baradaran Rahmanian, Navid Mader, Ilija Djordjevic, Thorsten Wahlers

**Affiliations:** 1 Department of Cardiothoracic Surgery, Heart Centre, Helios Hospital Siegburg, 53721 Siegburg, Germany; 2 Department of Cardiothoracic Surgery, Heart Centre, University Hospital Cologne, 50937 Cologne, Germany; 3 Department of Cardiothoracic Surgery, Helios University Hospital Wuppertal, University Witten/Herdecke, 42117 Wuppertal, Germany; 4 Department of Cardiothoracic Surgery, University Hospital Tuebingen, 72076 Tuebingen, Germany

**Keywords:** ECMO, cardiogenic shock, cardiac surgery, elective, emergent

## Abstract

The outcomes of patients with PCS and following ECMO therapy are associated with several preoperative risk factors. Our aim was to compare clinical presentation, ECMO-related data and in-hospital outcomes of patients treated with ECMO due to PCS after cardiac surgery, in regard to elective or emergent cardiac surgery procedures. Between April 2006 and October 2016, 164 consecutive patients that received VA-ECMO therapy due to PCS were identified and included in this retrospective cohort study. The patients were divided into groups based on the urgency of the initial procedures performed: elective group (ELG; *n* = 95) and an emergency group (EMG; *n* = 69). To compare the unequal patient groups, a propensity score-based matching (PSM) was applied (ELG, *n* = 56 vs. EMG, *n* = 56). The EMG primarily received ECMO intraoperatively (*p ≤* 0.001). In contrast, the ELG were needed ECMO support more frequently postoperatively (*p* < 0.001). In-hospital mortality accounted for 71% (*n* = 40) in the ELG and 76% (*n* = 43) in the EMG (*p* = 0.518). Outcome data showed no major differences in the (abdominal ischemia (*p* = 0.371); septic shock (*p* = 0.393): rhythm disturbances (*p* = 0.575); emergency re-thoracotomy (*p* = 0.418)) between the groups. The urgency of the initial procedures performed is secondary in patients suffering PCS and following ECMO. In this regard, PCS itself seems to trigger outcomes in cardiac surgery ECMO patients substantially.

## 1. Introduction

A patient’s preoperative hemodynamic state significantly impacts their mortality rates and outcomes after performed cardiac surgery. Elective coronary artery bypass graft surgery (CABG) is associated with a perioperative/hospital-mortality rate of approximately 3% [1,2], whereas CABG in acute myocardial infarction (MI) showed mortality rates up to 30% [3,4]. Similar differentiation is described in aortic valve surgery [5,6]. In this regard, the perioperative risk is highly depending on co-morbidities, postoperative complications, the hospital volume of specific procedures and the expertise of the involved physicians [7,8].

Alongside the impact of elective or emergent surgical procedures, postcardiotomy cardiogenic shock (PCS) is a complication associated with high mortality rates, especially in patients who require mechanical circulatory support (MCS) with extracorporeal membrane oxygenation (ECMO) [9,10]. However, refractory PCS requiring ECMO is described by an incidence of 0.5–1.5% [11,12].

Veno-arterial extracorporeal membrane oxygenation (VA-ECMO) is an increasingly used method for circulatory support [13]. VA-ECMO in postcardiotomy cardiogenic shock facilitates the improvement of hemodynamic status and significant increase in tissue perfusion [14]. Despite ongoing research, survival following VA-ECMO therapy remains low [14,15].

In the context of known distinctions of elective and emergent cardiac surgery procedures and their impact on perioperative results, the question arises whether outcomes of patients that require VA-ECMO support differ depending on the degree of emergency of the procedures performed. Therefore, the objective of this study was to compare clinical presentation, VA-ECMO-related data and in-hospital outcomes of patients treated with VA-ECMO due to PCS after cardiac surgery in regard to elective or emergent cardiac surgery.

## 2. Materials and Methods

**Figure 1:**
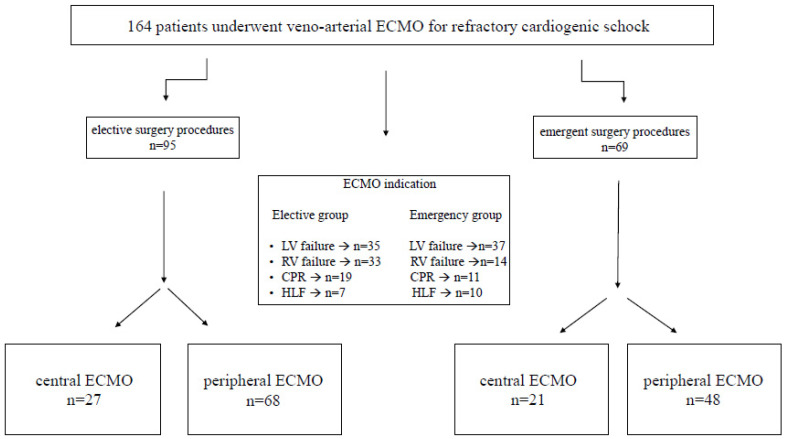
Flow chart illustrating patient selection and ECMO indication. ECMO: extracorporeal membrane oxygenation.

**Figure 2:**
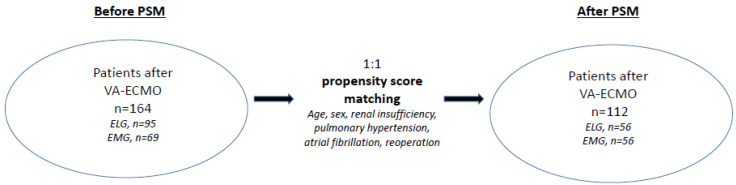
Study population of patients after VA-ECMO implantation. PSM: propensity score matching; ELG: elective group; EMG: emergency group.

The study was designed as a retrospective single center cohort analysis. Between April 2006 and October 2016, 164 consecutive patients with veno-arterial ECMO therapy due to PCS were identified and included in this study. To analyse the impact of urgency, patients were divided regarding the urgency of procedures performed in an elective group (ELG, *n* = 95) and an emergency group (EMG, *n* = 69). ECMO indications for both groups were: left ventricular (LV) failure, right ventricular (RV) failure, combined heart and lung failure, and cardiopulmonary resuscitation (CPR) (Figure 1). To compare the unequal patient groups a propensity score-based matching (PSM) was applied (Figure 2).

### 2.1. ECMO-Center Protocol

VA-ECMO support (Rotaflow, Maquet, Rastatt, Germany) was established in the case of inefficient weaning from cardio-pulmonary bypass (CPB) or as an ultima-ratio therapeutic option in the case of therapy refractory postcardiotomy cardiogenic shock after performed cardiac surgery procedures. Central VA-ECMO implantation was performed by switching inserted CPB cannulas from the right atrium and ascending aorta into the ECMO system. The thorax was packed and left opened. In case of already closed-chest, peripheral VA-ECMO was implanted through groin vessels using the percutaneous Seldinger’s technique.

Our anticoagulant protocol aimed towards an activated clotting time (ACT) 160–180 s and activated partial thromboplastin time (aPTT), 60–80 s after intravenous infusion of unfractionated heparin, to avoid potential thromboembolic events. Following this, echocardiography, laboratory parameters and chest X-rays were performed to evaluate the hemodynamic stability and possible weaning ability. Moreover, heart function was evaluated daily using transesophageal echocardiography (TEE). 

VA-ECMO weaning was initialized after haemodynamic stabilization. The ECMO flow rate was decreased, 100–200 mL/h. Moreover, lactate and urine outputs were assessed hourly. ECMO removal was feasible when TEE showed partial or full recovery under 2.0 L/min ECMO support, without increasing lactate concentration in the blood and decreasing urine output. All patients assumed to be suitable for weaning underwent surgical explanation of ECMO cannulas.

### 2.2. Data Collection

All relevant data were analyzed retrospectively, after extraction from our institutional database. The variables evaluated included:

- patients’ demographic characteristics (age, body mass index (BMI), sex, European System for Cardiac Operative Risk Evaluation (EuroSCORE II), Society of Thoracic Surgeons (STS Score));
- patients’ status before ECMO support (renal insufficiency with dialysis, previous heart surgery, catecholamine therapy, left-ventricular ejection fraction (LV-EF));
- laboratory parameter (creatine kinases (CK), creatine kinases muscle brain (CK-MB), glutamate-oxalacetate transaminase (GOT), glutamate-oxalacetate transaminase (GLT), bilirubin);
- implantation data (ECMO duration, ECMO complications, concomitant intra-aortic balloon pump (IABP) implantation, ECMO weaning);
- early outcome data (in-hospital all-cause mortality, intensive care unit (ICU)- and hospital stay, renal failure requiring dialysis, disabling cerebrovascular events, septic shock, emergency re-thoracotomy, abdominal ischemia, rhythm disturbances, tracheotomy and red blood cell transfusion rate).

### 2.3. Endpoints of the Study

The primary endpoint was in-hospital all-cause mortality. The secondary outcome parameters were: in-hospital stay, renal failure requiring dialysis (glomerular filtration rate (GFR) < 15 mL/min, life-threatening hyperkalemia, refractory acidosis and hypervolemia causing end-organ complications), disabling cerebrovascular events (ischemic stroke or hemorrhagic stroke), septic shock (persistent hypotension requiring vasopressors to maintain mean arterial pressure of 65 mm/Hg or higher and a serum lactate level greater than 2 mmol/L despite adequate volume resuscitation), emergency re-thoracotomy (blood loss with a hemoglobin decrease of greater than 3 g/dL or any hemoglobin decrease of greater than 4 g/dL), abdominal ischemia (detected with computed tomography (CT) angiography), rhythm disturbances, tracheotomy and red blood cell transfusion rate. Early outcomes were analyzed for both cohorts (ELG and EMG).

### 2.4. Ethics

The study was conducted in accordance with the Declaration of Helsinki (as revised in 2013). The Ethics Committee of the Medical Faculty of the University of Cologne stated that we are exempted from applying for ethical approval as no separate ethics application or statement of ethical approval by the local ethics committee are required for performing purely retrospective clinical studies under German law.

### 2.5. Statistical Analysis

Statistical analysis was performed using the Statistical Package for Social Sciences, version 23.0 (SPSS IBM, Chicago, IL, USA). All data were presented as continuous or categorical variables. Categorical data were expressed as total numbers and percentages. Continuous data were evaluated for normality using a one-sample Kolmogorov-Smirnov test and were expressed as the mean ± standard deviation (SD) in cases of normally distributed, or median (interquartile range) in cases of non-normally distributed continuous variables. Univariate analysis was performed using either a Student t or a Mann-Whitney U test for normally and non- normally distributed continuous variables, respectively. Pearson’s χ² or Fisher exact tests were used for the comparison of categorical data, depending on the minimum expected count in each cross-tab. Logistical regression was conducted in order to create the predicted variable. A rigorous 1:1 nearest neighbor-matching algorithm, without replacement, was used with a 0.2 caliper set. Standardized-mean-differences (d-values) were calculated, and absolute d-values under 0.2 were considered to be an indicator of adequate balance and sufficient reduction of bias; *p* values < 0.05 were considered statistically significant.

## 3. Results

### 3.1. Preoperative Profile Differences Depending on Urgency before and after PSM

The preoperative characteristics of the study population are presented in Table 1. Both groups differed in several preoperative characteristics. Congruent to the preoperative co-morbidity profile, the EUROSCORE II (*p* < 0.001 before PSM and *p* < 0.001 after PSM) and the STS score (*p* = 0.001 before PSM and *p* < 0.001 after PSM) were significantly higher in the EMG. Preoperative catecholamine therapy (*p* < 0.001 before PSM *p* < 0.001 after PSM) was significantly higher in the EMG compared to the ELG. Similarly, duration of mechanical ventilation (*p* < 0.001 before PSM *p* < 0.001 after PSM) was significantly higher in the EMG. 

### 3.2. Time Point and Indication of ECMO Therapy as Major Characteristics of Urgency Procedures after PSM

The ECMO-related parameters are summarized in Table 2. The EMG primarily received ECMO intraoperatively (ELG *n* = 21 (38%) vs. EMG *n* = 45 (80%), *p ≤* 0.001). In contrast, the ELG required ECMO support more frequently postoperatively (ELG *n* = 35 (62%) vs. EMG *n* = 11 (20%), *p* < 0.001). The type of ECMO support, central (ELG *n* = 14 (25%) vs. EMG *n* = 8 (14%), *p* = 0.154) or peripheral (ELG *n* = 42 (75%) vs. EMG *n* = 48 (86%), *p* = 0.234), did not differ between the groups. 

### 3.3. Intra- and Postoperative Outcomes of Patients with PCS and ECMO Therapy after PSM

Intraoperative and postoperative data, before and after PSM, is presented in Table 3. CABG was performed significantly more often in the EMG (ELG *n* = 39 (69%) vs. EMG *n*= 49 (87%); *p* = 0.037). In-hospital mortality accounted for 71% (*n* = 40) in the ELG and 76% (*n* = 43) in EMG (*p* = 0.518). The outcome data showed no major differences (abdominal ischemia (*p* = 0.371); septic shock (*p* = 0.393): rhythm disturbances (*p* = 0.575); emergency re-thoracotomy (*p* = 0.418) between the groups. 

## 4. Discussion

The analysis performed clearly showed that the preoperative risk profile significantly differed between the compared patient cohorts, as expected. While the electively operated patients were treated with ECMO after surgery, the emergently operated patients showed a failure to be weaned from cardio-pulmonary bypass more frequently and required ECMO support intraoperatively. Despite significant distinctions in the preoperative, intraoperative and ECMO-related parameters, the outcome data were comparable, resulting in high mortality (70–80%) in both groups.

### 4.1. Risk Stratification of Urgency in Treatment Strategies

In general, urgency is known to impact the outcomes of patients [16]. Several studies have conducted comparative investigations in regard to elective or emergent surgical procedures performed [17,18,19]. De Rango and colleagues, in their retrospective analysis of 141 patients, showed that in-hospital mortality was strongly associated with urgency in thoracic aortic endovascular repair [20].The results from other disciplines are not generalizable for cardiac surgery patients. Kabahizi and colleagues provided recent data of 1157 patients, indicating that urgent transcatheter aortic valve replacement (TAVR) might be performed at a similar risk to elective TAVR [21]. Singh and colleagues postulated that the emergent use of mechanical circulatory support during TAVR is associated with high short- and long-term mortality in comparison to the elective application [22]. In particular, specific analysis of cardiac surgery procedures regarding urgency is scarce, particularly in terms of ECMO support [23,24,25]. Data in this specific field is lacking and our analysis is one of the first to investigate the impact of urgency on outcomes after ECMO therapy due to PCS [26,27,28].

### 4.2. Urgency Is Secondary in Cardiac Surgery ECMO Patients

In our analysis, the direct comparison between patients divided into groups based on urgency showed that in- hospital mortality rates were comparable between the groups. Despite a significantly higher EUROSCORE II and STS score, the outcomes were not different. This fact clearly indicates that PCS after cardiac surgery diminishes the presumed beneficial outcomes of patients that have had elective cardiac surgery in comparison to emergency procedures [23]. Independently of several risk factors, the leading cause of limited survival in cardiac surgery ECMO patients might be substantially triggered by PCS itself [29,30,31,32,33]. Badulak corroborates this postulate, suggesting that increased morbidity and mortality following ECMO insertion is independent to preoperative co-morbidities [34]. In fact, both mortality rates from our analysis (78% ELG vs. 71% EMG; *p* = 0.315) match the mortality described in the current literature [35]. Therefore, therapeutic strategies in ECMO patients may not be influenced by the initial procedure [36].

### 4.3. Operative Characteristics in ECMO Patients Depending on Urgency

As acute CABG procedures present a significant proportion of emergent cardiac surgery procedures, it is not surprising that CABG was performed significantly more often in the EMG in our analysis. Moreover, emergent procedure patients showed higher cardio-pulmonary bypass and cross clamp time, necessitating a higher number of patients in need of ECMO support intraoperatively, in comparison to elective procedure patients. The fact that patients in the EMG were provided long-term assist devices systems significantly more often remains unclear from our data and might be the focus of further investigation.

### 4.4. Study Limitations

The retrospective non-randomized design and analysis of a limited number of patients from a single center reduces the statistical power of the study. Therefore, the presented data should be taken with caution. However, the present report focused on short-term outcomes and did not evaluate long-term results and quality of life measures. Moreover, the collection of data was restricted to the variables that were available in electronic or written patient notes and flowcharts.

## 5. Conclusions

Urgency impacts the incidence of PCS and subsequent ECMO therapy in cardiac surgery patients, however, urgency itself is secondary in patients suffering PCS and following ECMO therapy, according to our data. In this regard, PCS itself seems to trigger outcomes in cardiac surgery ECMO patients substantially. In communication with relatives, this fact might help physicians to evaluate, categorize and predict outcomes among patients with PCS and consecutive ECMO therapy.

## Figures and Tables

**Table 1:** Preoperative characteristics of patients with PCS and ECMO-therapy before and after PSM.

Before PSM				After PSM		
Preoperative Characteristics	Elective (*n* = 95)	Emergency (*n* = 69)	*p*-Value	Elective (*n* = 56)	Emergency (*n* = 56)	*p*-Value
Age, years, mean (min/max)	69 (58/73)	61 (54/68)	0.001	62 (58/66)	62 (59/64)	0.981
Body mass index, kg/m², mean (min/max)	27 (25/32)	26 (24/29)	0.321	28 (26/45)	26 (25/27)	0.139
Female gender, (*n*) %	31 (33)	14 (20)	0.080	19 (33.9)	10 (17.9)	0.052
Chronic lung disease, (*n*) %	11 (12)	8 (12)	0.963	4 (7.1)	7 (12.5)	0.341
Pulmonary hypertension, (*n*) %	24 (25)	2 (2.9)	<0.001	4 (7.1)	2 (3.6)	0.679
Renal insufficiency, (*n*) %	32 (34)	12 (1.4)	0.024	13 (23.2)	12 (21.4)	0.820
Renal insufficiency with dialysis, (*n*) %	5 (5.3)	2 (2.9)	0.701	3 (5.4)	2 (3.6)	0.647
Hypertension, (*n*) %	71 (75)	43 (62)	0.147	40 (71.4)	36 (64.3)	0.418
Diabetes mellitus, (*n*) %	33 (35)	17 (25)	0.204	17 (32.1)	15 (26.8)	0.534
Peripheral vascular disease, (*n*) %	15 (16)	14 (20)	0.404	5 (8.9)	12 (21.4)	0.065
Atrial fibrillation, (*n*) %	35 (37)	8 (12)	<0.001	12 (21.4)	8 (14.3)	0.324
Catecholamine therapy, (*n*) %	0 (0.0)	45 (65)	<0.001	0 (0.0)	36 (65.5)	<0.001
Mechanical ventilation, (*n*) %	0 (0.0)	38 (55)	<0.001	0 (0.0)	31 (55.4)	<0.001
Left-ventricular ejection-fraction, mean (min/max)	50(39/60)	35(20/50)	<0.001	50 (45/55)	33 (28/38)	<0.001
Lactate, mmol/L, mean (min/max)	7.4(4.7/13.1)	10.8(5.4/14.8)	0.025	8.7 (7.0/10.3)	11.1 (9.2/12.9)	0.053
EuroSCORE II, %, mean (min/max)	7.0(4.8/9.0)	12.0(11.0/15.0)	<0.001	6.0 (5.2/6.8)	12.7 (11.9/13.5)	<0.001
STS Score, mean (min/max)	3.7(1.5;6.5)	17.7 (5.9/28.4)	0.001	2.9 (1.8/4.0)	19.3 (12.6/25.9)	<0.001

Data is expressed as median (range) or percentage (counts) as indicated. IABP = intra-aortic balloon pump, ECMO = extracorporeal membrane oxygenation, CK = creatine kinases, CK-MB = creatine kinases muscle-brain.

**Table 2:** ECMO-related data after PSM.

ECMO Related Data	Elective (*n* = 56)	Emergency (*n* = 56)	*p*-Value
ECMO implantation intraoperative, (*n*) %	21 (38)	45 (80)	<0.001
ECMO implantation postoperative, (*n*) %	35 (62)	11 (20)	<0.001
central ECMO, (*n*) %	14 (25)	8 (14)	0.154
peripheral ECMO, (*n*) %	42 (75)	48 (86)	0.234
ECMO indication			
Left ventricular failure, (*n*) %	23 (41)	31 (55)	0.185
Right ventricular failure, (*n*) %	12 (22)	15 (27)	0.658
Combined heart and lung failure, (*n*) %	6 (10)	4 (8)	0.740
Cardiopulmonary resuscitation, (*n*) %	15 (27)	6 (10)	0.052
ECMO outcome			
Duration, hours, mean (min/max)	81 (64/97)	91 (72/109)	0.408
ECMO complication, (*n*) %	36 (64.3)	32 (57)	0.439
ECMO local complication, (*n*) %	10 (17.9)	13 (23)	0.320
IABP implantation, (*n*) %	45 (80.4)	48 (85)	0.308
ECMO weaning, (*n*) %	32 (57.1)	26 (46.4)	0.257

Data is expressed as median (range) or percentage (counts) as indicated. ECMO = extracorporeal membrane oxygenation, IABP = intra-aortic balloon pump.

**Table 3:** Intra- and postoperative outcomes of patients with postcardiotomy cardiogenic shock and ECMO-therapy after PSM.

Intra- and Postoperative Outcomes	Elective (*n* = 56)	Emergency (*n* = 56)	*p*-Value
CABG, (*n*) %	39 (69)	49 (87)	0.037
CABG, isolated, (*n*) %	22 (39)	39 (69)	0.002
Aortic valve replacement, isolated, (*n*) %	5 (8.9)	1 (1.8)	0.206
Other valve surgery, (*n*) %	2 (3.6)	0 (0.0)	0.495
Cardiopulmonary bypass time, minutes, mean (min/max)	176 (149/203)	176 (145/187)	0.345
Aortic cross-clamp time, minutes, mean (min/max)	78 (64/92)	60 (47/68)	0.057
Postoperative outcomes			
In hospital all-cause mortality, (*n*) %	40 (71)	43 (76)	0.518
Disabling cerebrovascular events, (*n*) %	12 (21)	15 (26)	0.330
Abdominal ischemia, (*n*) %	11 (19)	15 (26)	0.371
-Emergency laparotomy, (*n*) %	4 (7.1)	4 (7.1)	0.642
Septic shock, (*n*) %	8 (15)	12 (21)	0.393
Rhythm disturbances, (*n*) %	29 (51)	29 (51)	0.575
Emergency re-thoracotomy, (*n*) %	40 (71)	36 (64)	0.418
Dialysis, (*n*) %	35 (63)	36 (66)	0.448
Mechanical ventilation, days	10 (8/12)	16 (12/18)	0.056
Tracheotomy, (*n*) %	13 (23.2)	24 (42.9)	0.022
Red blood cell transfusion, units, mean (min/max)	33 (27/38)	32 (22/49)	0.093
Length of ICU stay, days, mean (min/max)	11 (8/13)	20 (11/29)	0.051
Discharge out of hospital, (*n*) %	13 (23.2)	16 (28.6)	0.518
Postoperative laboratory parameters			
CK maximum, U/L, mean (min/max)	1740 (1007/2473)	2361 (1644/3078)	0.227
CK-MB maximum, U/L, mean (min/max)	168 (96/240)	267 (179/355)	0.083
Bilirubin, mg/dL, mean (min/max)	3.2 (2.8/5.3)	6.2 (3.1/7.9)	0.178
GOT maximum, U/L, mean (min/max)	752 (252/1898)	1068 (315/3785)	0.071
GPT maximum, U/L, mean (min/max)	295 (125/590)	596 (225/1875)	0.338

Data is expressed as median (range) or percentage (counts) as indicated. CABG = coronary artery bypass grafting, CK = creatine kinases, CK-MB = creatine kinases muscle-brain, ICU = intensive care unit, GOT = glutamate-oxalacetate transaminase, GPT = glutamate-pyruvate transaminase.

## Data Availability

Data is available on a special request.
